# The association between graded prognostic assessment and the prognosis of brain metastases after whole brain radiotherapy: a meta-analysis

**DOI:** 10.3389/fonc.2023.1322262

**Published:** 2024-01-09

**Authors:** Xiaohan Geng, Changgui Kou, Jianfeng Wang

**Affiliations:** ^1^ Department of Epidemiology and Biostatistics, School of Public Health, Jilin University, Changchun, China; ^2^ Department of Radiotherapy, China-Japan Union Hospital, Jilin University, Changchun, China

**Keywords:** brain metastasis, whole brain radiotherapy, graded prognostic assessment, prognosis, meta-analysis

## Abstract

**Introduction:**

This meta-analysis aims to provide evidence-based medical evidence for formulating rational treatment strategies and evaluating the prognosis of brain metastasis (BM) patients by assessing the effectiveness of the graded prognostic assessment (GPA) model in predicting the survival prognosis of patients with BM after whole-brain radiotherapy (WBRT).

**Methods:**

A comprehensive search was conducted in multiple databases, including the China Biomedical Literature Database (CBM), China National Knowledge Infrastructure (CNKI), PubMed, Wanfang database, Cochrane Library, Web of Science, and Embase. Cohort studies that met the inclusion and exclusion criteria were selected. The quality of the included literature was evaluated using the Newcastle-Ottawa Scale, and all statistical analyses were performed with R version 4.2.2. The effect size (ES) was measured by the hazard ratio (HR) of overall survival (OS). The OS rates at 3, 6, 12, and 24 months of patients with BM were compared between those with GPAs of 1.5–2.5, 3.0, and 3.5–4.0 and those with GPAs of 0–1 after WBRT.

**Results:**

A total of 1,797 participants who underwent WBRT were included in this study. The meta-analysis revealed a significant association between GPA and OS rates after WBRT: compared with BM patients with GPA of 0–1, 3-month OS rates after WBRT were significantly higher in BM patients with GPA of 1.5–2.5 (HR = 0.48; 95% CI: 0.40–0.59), GPA of 3 (HR = 0.38; 95% CI: 0.25–0.57), and GPA of 3.5–4 (HR = 0.28; 95% CI: 0.15–0.52); 6-month OS rates after WBRT were significantly higher in BM patients with GPA of 1.5–2.5 (HR = 0.48; 95% CI: 0.41–0.56), GPA of 3 (HR = 0.33; 95% CI: 0.24–0.45), and GPA of 3.5–4 (HR = 0.24; 95% CI: 0.16–0.35); 12-month OS rates after WBRT were significantly higher in BM patients with GPA of 1.5–2.5 (HR = 0.49; 95% CI: 0.41–0.58), GPA of 3 (HR = 0.48; 95% CI: 0.32–0.73), and GPA of 3.5–4 (HR = 0.31; 95% CI: 0.12–0.79); and 24-month OS rates after WBRT were significantly higher in BM patients with GPA of 1.5–2.5 (HR = 0.49; 95% CI: 0.42–0.58), GPA of 3 (HR = 0.49; 95% CI: 0.32–0.74), and GPA of 3.5–4 (HR = 0.38; 95% CI: 0.15–0.94).

**Conclusion:**

BM patients with higher GPAs generally exhibited better prognoses and survival outcomes after WBRT compared to those with lower GPAs.

**Systematic review registration:**

https://www.crd.york.ac.uk/prospero/, identifier CRD42023422914.

## Introduction

In recent years, brain metastasis (BM) has become one of the most common malignant tumors in adults (1). BM occurs when tumor cells from other parts of the body spread to the central nervous system through the bloodstream (2). It has been observed that over 25% of patients with malignant tumors develop metastases in the process of disease progression (3). Due to advancements in clinical imaging techniques, improved tumor treatments, increased patient survival times, and an aging population, the incidence and clinical detection rates of BM are increasing year by year (4–10). Consequently, the number of patients with BM is increasing, and they often experience poor prognosis and high mortality rates (11). Clinically, the emergence of BM has become a new challenge for oncologists (12, 13). The prognosis of BM patients might vary based on the origin of the primary tumor and important prognostic factors, which have attracted more and more attention (14).

Various primary disease types can lead to BM (15). Studies have shown that lung cancer is the most common primary source of BM (16), and more than half of the pathological types are small cell lung cancer (SCLC) (17). Breast cancer, as the second major tissue source of BM (18), is most commonly seen in ER-negative and HER2-positive patients (19). Malignant melanoma is the third most common lesion of BM (20). Additionally, there has been an increasing trend in the incidence of BM originating from kidney and colorectal cancer in recent years (21). Clinically, the presence of BM in the brain can cause compression of brain tissue at the site of tumor formation and an increase in intracranial pressure, leading to various neurological symptoms associated with BM, such as dizziness, headache, nausea, vomiting, epileptic seizures, impaired vision, and language difficulties (22). In severe cases, it can even result in a stroke (23).

Surgery, whole-brain radiotherapy (WBRT), stereotactic radiotherapy (SRT), and chemotherapy are the main therapeutic options currently available for BM patients (24). Surgery is not the treatment of choice for most BM patients due to its high disability rate and risk of recurrence (25–27). However, it is suitable for patients with an unidentified primary tumor, single metastasis, large intracranial lesion, or those experiencing neurological symptoms caused by associated vasogenic edema and mass effect (28). In clinical practice, WBRT and stereotactic radiosurgery (SRS) are the main radiotherapy modalities, and the most basic radiotherapy method is WBRT (27). WBRT is widely used in the treatment of multiple metastases due to its significant palliative effect on tumors (25). Despite its long history, WBRT remains an important treatment option in the current stage of clinical practice (29, 30). In recent years, the combination of WBRT with targeted therapy, or SRS, has also generated substantial interest and discussions in clinical practice (31).

The classification of prognostic assessment of patients with BM has a certain guiding value for clinical management, treatment selection, and stratification of clinical trials (32). Prognostic indices play a crucial role in BM radiotherapy by providing valuable information to guide patient decision-making and clinical trial stratification (33). Over time, various classification models for prognostic assessment have evolved and developed, but there is still no unified standard scheme. These models integrate multiple prognostic factors associated with the survival of BM patients, continuously evolving and developing (34–42). In 2008, a new prognostic prediction model called the graded prognostic assessment (GPA) was proposed (38). Sperduto’s study (43) demonstrated a positive correlation between GPA scores and prognosis. Additionally, it was found that the median survival time of patients with different GPA scores (0–1, 1.5–2.5, 3, and 3.5–4.0) showed a progressive increase, and the median survival time of patients in each GPA group was significantly different. As one of the widely used classification criteria for BM patients undergoing radiotherapy in clinical practice, the GPA model is characterized by its strong quantitative nature and ease of memorization. It serves as the most standardized, objective, and user-friendly indicator for assessing the prognosis of BM patients (44). In a study conducted by Viani et al. (45), different prognostic indices were compared using a neural network-based approach, and it was found that GPA had better predictive performance for the prognosis of BM patients undergoing WBRT (with or without neurosurgery) compared to several other indices.

The purpose of this study is to investigate the effectiveness of the GPA model in predicting the prognosis of BM patients treated with WBRT and explore the relationship between GPA and patients’ prognosis. The ultimate goal is to obtain more objective and reliable findings and provide high-quality evidence-based medical evidence for the prognostic assessment of BM patients undergoing WBRT.

## Materials and methods

This meta-analysis was conducted and reported according to the protocol outlined by Preferred Reporting Items for Systematic Reviews and Meta-Analyses (PRISMA) using a research question framed by PICOS methodology (46) and it was registered in the PROSPERO International Prospective Register of Systematic Reviews (CRD42023422914). Ethical approval and informed consent were not required, as this study involved secondary research based on previously published data.

### Systematic literature search

Two researchers independently conducted comprehensive searches in the following databases: China National Knowledge Infrastructure (CNKI), PubMed, Wanfang database, Cochrane Library, Web of Science, Embase, and China Biomedical Literature Database (CBM). The search covered the time period from the inception of the databases to 31 December 2022. The following search terms were used in order to find the desired studies: (“metastasis OR metastases OR metastatically OR metastatics OR metastatization OR metastatize OR metastatized OR metastatizing OR secondary OR metastatic” OR “brain metastasis OR brain metastases OR metastatic brain tumor OR cerebral metastases OR Central Nervous System Metastasis OR brain neoplasms”) AND (“GPA OR Graded Prognostic Assessment”) AND (“whole brain radiotherapy OR WBRT”).

### Inclusion and exclusion criteria

The inclusion criteria for the selection of appropriate studies were defined according to the PI(E)COS approach: Population: patients diagnosed with brain metastatic tumors and treated with WBRT; Exposure: GPA; Controls: patients with the lowest GPA; Outcomes: overall survival (OS) at 3, 6, 12, and 24 months after WBRT for patients with BM, with the effect size reported as hazard ratio (HR); Study design: cohort study.

The exclusion criteria were as follows: Literature such as reviews, surveys, case reports, expert opinions, conference abstracts, and animal studies; Invalid or incomplete article data; Studies with the same patient populations; Studies without a reasonable and appropriate trial design.

### Literature screening and quality assessment

The retrieved literature was screened based on the inclusion and exclusion criteria. Relevant information such as authors, country, year, sex ratio, treatment method, 3-, 6-, 12-, and 24-month OS rates, and HRs were extracted and tabulated. In cases where the required data could not be directly obtained from the original text, Tierney’s method was applied to extract the necessary information (47). The process of literature screening, data extraction, and quality assessment was independently conducted by two researchers, and in case of any disagreement, consensus was reached through discussions with experts to minimize potential selection bias and information bias.

The quality assessment of the included literature was conducted using the Newcastle-Ottawa Scale (NOS), which assigned scores in eight areas, including the investigation and assessment of the exposure group. A score of ≥6 was considered indicative of good quality.

### Statistical analysis

The data were analyzed using R version 4.2.2. The control group consisted of BM patients with a GPA of 0–1. To combine the HR of each outcome index and its 95% confidence interval (CI), we selected a fixed-effects model or random-effects model based on the results of the *I* ^2^ test. The random-effects model was chosen when significant heterogeneity was observed among the included studies (*I* ^2^ ≥ 50%), while the fixed-effects model was used when the heterogeneity was not significant (*I* ^2^ < 50%). The sensitivity analysis was conducted to assess the stability of the primary combined results and to identify potential sources of bias. The assessment of potential publication bias was conducted by combining funnel plots and Egger’s test (48). An asymmetric funnel plot with a *p*-value of less than 0.05 was considered to indicate significant publication bias. If potential publication bias is detected, the combined results will be adjusted using the trim and filling method to analyze the impact of publication bias on the merged results.

## Results

### Literature search and study selection

**Figure 1:**
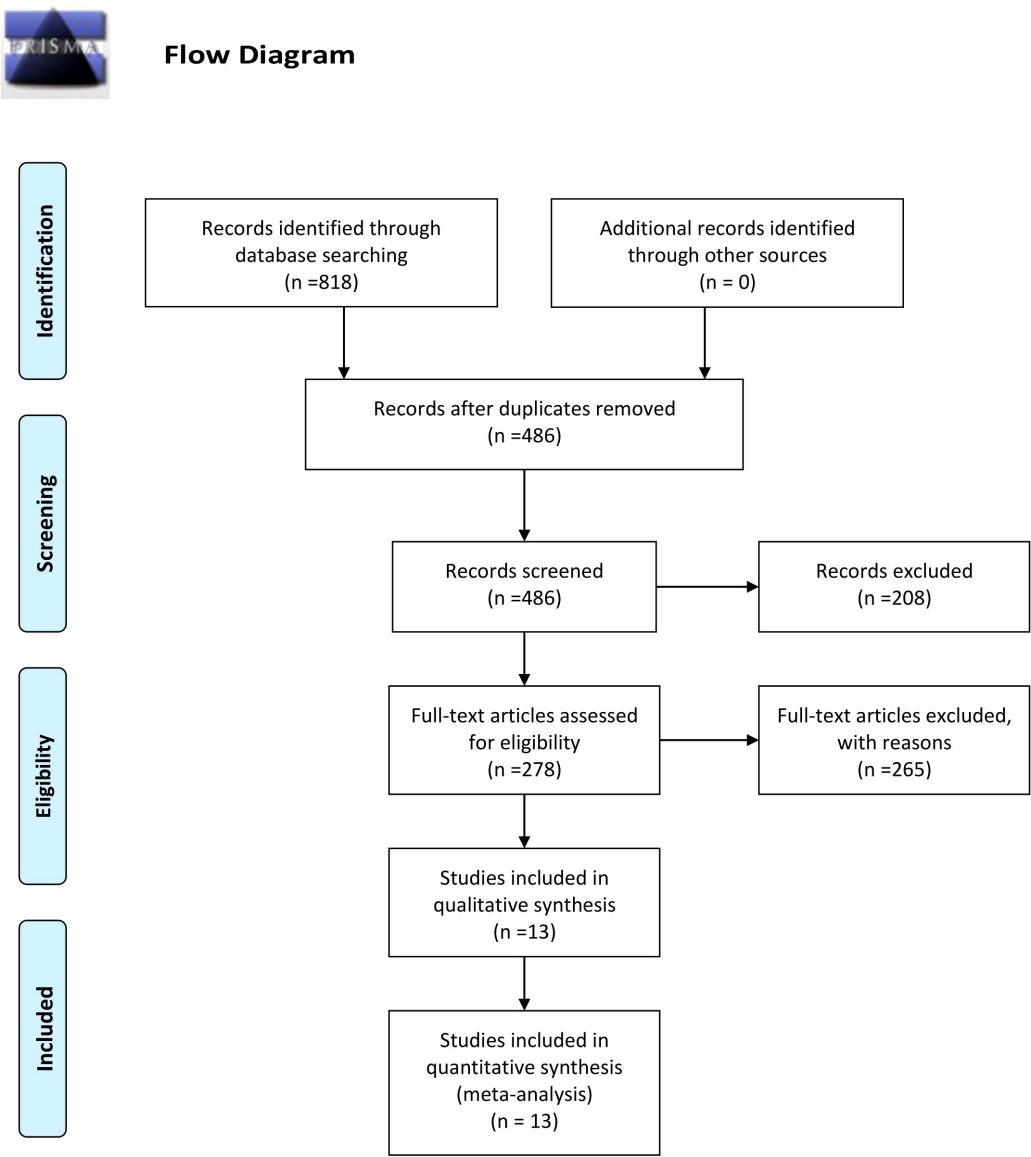
Flowchart of study selection.

The detailed process of literature screening is depicted in **Figure 1**. As shown in the flow chart, 332 duplicates were eliminated using EndNote 20.1 software and manual examination initially. Subsequently, the titles and abstracts of the remaining articles were carefully reviewed to assess their relevance to the topic, leading to the exclusion of 208 studies. Furthermore, a thorough examination of the full text allowed the exclusion of 265 studies based on the predetermined inclusion and exclusion criteria. Ultimately, there were 13 cohort studies included in our study, six of which originated from China, four from Norway, and one each from Sweden, Germany, and France.

### Study characteristics

Two researchers independently conducted a detailed review and extracted basic information such as authors, countries, sex ratio, GPA distribution, and so on. The characteristics of the 13 studies, covering 1,797 participants, are summarized in **Table 1**.

**Table 1:** The basic characteristics of the included studies.

Author (year)	NOS score	Country	Sex (male/female)	Mean age	Patients with GPA 0–1/1.5–2.5/3/3.5–4	Primary tumors	Treatment	Outcome indicators		
								GPA 1.5–2.5 vs. 0–1	GPA 3 vs. 0–1	GPA 3.5–4 vs. 0–1
**Fan** (2014) (49)	**8**	**China**	**142/68**	**55**	**42/132/22/12**	**NSCLC**	**WBRT**	①②③④	②③④	**④**
**Li D** (2013) (50)	**8**	**China**	**122/64**	**56**	**50/43/41/52**	**LC, BC, MM, CRC, HCC, ESCA, STAD, GO, other**	**WBRT**	②	②	**②**
**Nieder1** (2009) (51)	**9**	**Norway**	**15/9**	**56**	**9/15/0/0**	**NSCLC, SCLC, BC, MM, RC, PC, UC**	**WBRT**	①②	**–**	**–**
**Richard** (2015) (52)	**8**	**Germany**	**148/86**	**64**	**95/69/36/16**	**MEL**	**WBRT**	①②③④	**①②③④**	**①②③**
**Georgios** (2017) (53)	**7**	**Sweden**	**131/149**	**66.6**	**168/98/–/–**	**SCLC, LUAD, LUSC, NOS/mixed type**	**WBRT**	①②③④	**–**	**–**
**Nieder2** (2009) (54)	**7**	**Norway**	**–/–**	**61**	**38/93/35/7**	**NSCLC**	**WBRT**	**①②**	**①②**	**①②**
**Hong** (2013) (55)	**9**	**China**	**28/24**	**58.5**	**9/31/–/–**	**NSCLC, SCLC**	**WBRT, WBRT + boost**	**②③**	**–**	**–**
**Tong** (2014) (56)	**7**	**China**	**80/29**	**54**	**25/66/11/7**	**LUAD, LUSC, other**	**WBRT**	**①②③④**	**–**	**–**
**Nieder3** (2008) (57)	**8**	**Norway**	**42/22**	**66**	**8/38/12/6**	**NSCLC, BC**	**WBRT**	**①②**	**①②**	**①②**
**Chen** (2021) (58)	**7**	**China**	**40/35**	**61**	**12/–/–/3**	**LUAD, LUSC, ASC, NSCLC, SCLC**	**WBRT**	**–**	**–**	**④**
**Delphine** (2013) (59)	**8**	**France**	**493/284**	**61.3**	**272/–/–/67**	**LC, BC, MEL, RCC, GI, other**	**WBRT**	**–**	**–**	**①②③④**
**Nieder4** (2009) (60)	**9**	**Norway**	**–/–**	**–**	**9/18/5/0**	**CLC**	**WBRT**	**①②**	**①②**	**–**
**Du** (2020) (61)	**8**	**China**	**90/54**	**59**	**45/80/–/–**	**SCLC, LUAD, LUSC**	**WBRT, WBRT + boost, SIB-IMRT**	**①②③④**	**–**	**–**

NSCLC, non-small cell lung cancer; LC, lung cancer; BC, breast cancer; MM, malignant melanoma; CRC, colorectal cancer; HCC, hepatocellular carcinoma; ESCA, esophageal carcinoma; STAD, stomach adenocarcinoma; GO, gynecological oncology; SCLC, small cell lung cancer; RC, rectal cancer; PC, prostate cancer; UC, uterine cancer; MEL, melanoma; LUAD, lung adenocarcinoma; LUSC, lung squamous cell carcinoma; NOS, NSCLC not otherwise specified; ASC, adenosquamous carcinoma; RCC, renal cell carcinoma; GI, gastrointestinal cancer; CLC, colorectal cancer.

①, 3-month OS; ②, 6-month OS; ③ 12-month OS; ④, 24-month OS; “–” unreported.

### Quality assessment of included literature

The included studies were assessed for quality using the NOS scale, and they all received scores greater than 6, indicating a relatively high level of evidence quality (**Table 1**).

## Results of meta-analysis

### Association analysis between GPA and 3-month OS after WBRT in patients with BM

**Figure 2:**
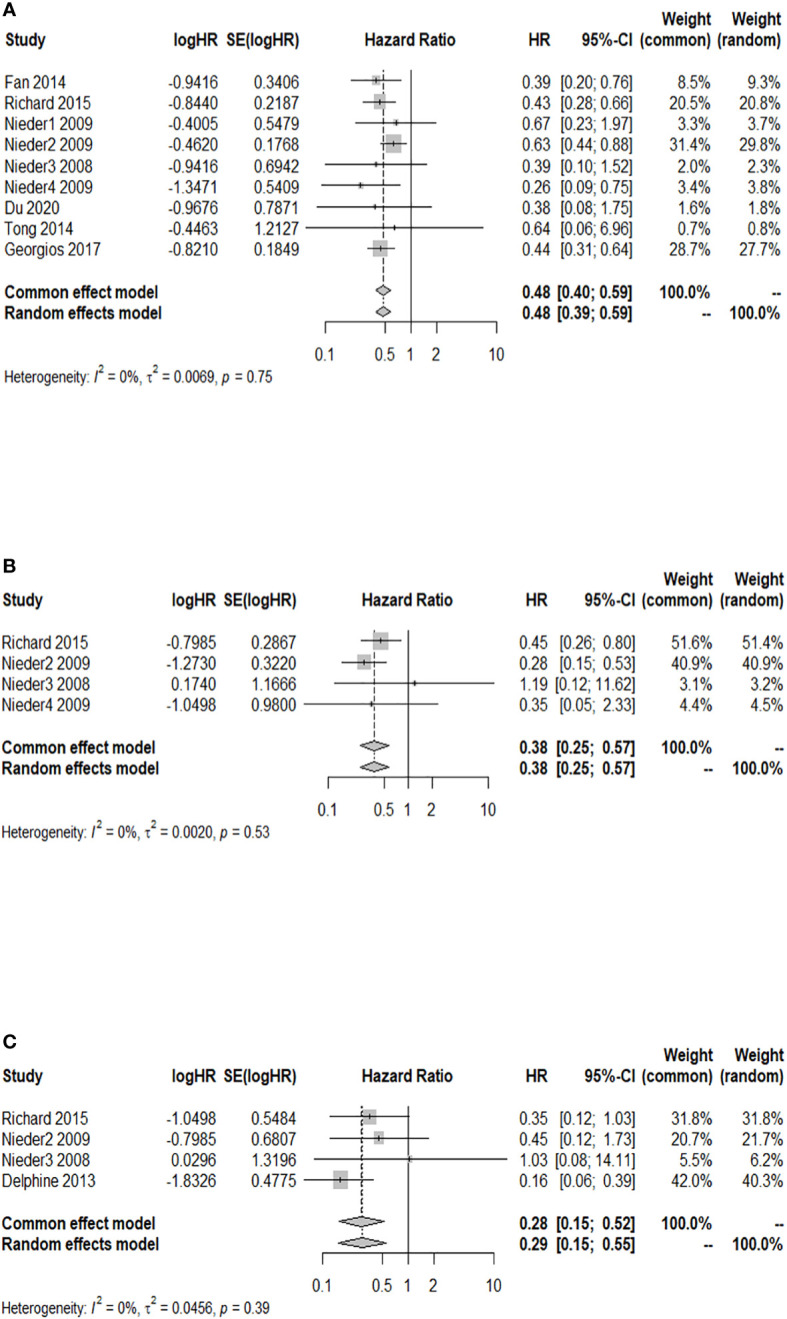
Forest plots of the association analysis between GPA and 3-month OS after WBRT in patients with BM. **(A)** Comparison of 3-month OS after WBRT in patients with BM of GPA 1.5–2.5 vs. 0–1. **(B)** Comparison of 3-month OS after WBRT in patients with BM of GPA 3 vs. 0–1. **(C)** Comparison of 3-month OS after WBRT in patients with BM of GPA 3.5–4 vs. 0–1.

A total of nine studies reported a 3-month OS rate after WBRT in patients with a GPA of 1.5–2.5, compared to those with a GPA of 0–1. There was no significant heterogeneity among them (*I* ^2 ^= 0.0%). The fixed-effects model was selected for the meta-analysis. As shown in **Figure 2A**, after WBRT, patients with a GPA of 1.5–2.5 had a significantly higher 3-month OS rate than patients in the GPA of 0–1 group (HR = 0.48; 95% CI: 0.40–0.59). Four studies provided a 3-month OS rate after WBRT in patients with a GPA of 3 compared to patients with a GPA of 0–1, and the *I* ^2^ test showed no significant heterogeneity among them (*I* ^2 ^= 0.0%), so the fixed-effects model was used for the combined analysis. The 3-month OS rate was significantly higher in patients with a GPA of 3 than in patients with a GPA of 0–1 (HR = 0.38; 95% CI: 0.25–0.57, **Figure 2B**). A total of four studies presented the 3-month OS rate after WBRT in patients with a GPA of 3.5–4 vs. those with a GPA of 0–1. The heterogeneity among these four cohort studies was not significant (*I* ^2 ^= 0.0%). The meta-analysis results indicated that the patients with a GPA of 3.5–4 had a significantly higher 3-month OS rate after WBRT compared to the GPA 0–1 group (**Figure 2C**: HR = 0.28; 95% CI: 0.15–0.52).

### Association analysis between GPA and 6-month OS after WBRT in patients with BM

**Figure 3:**
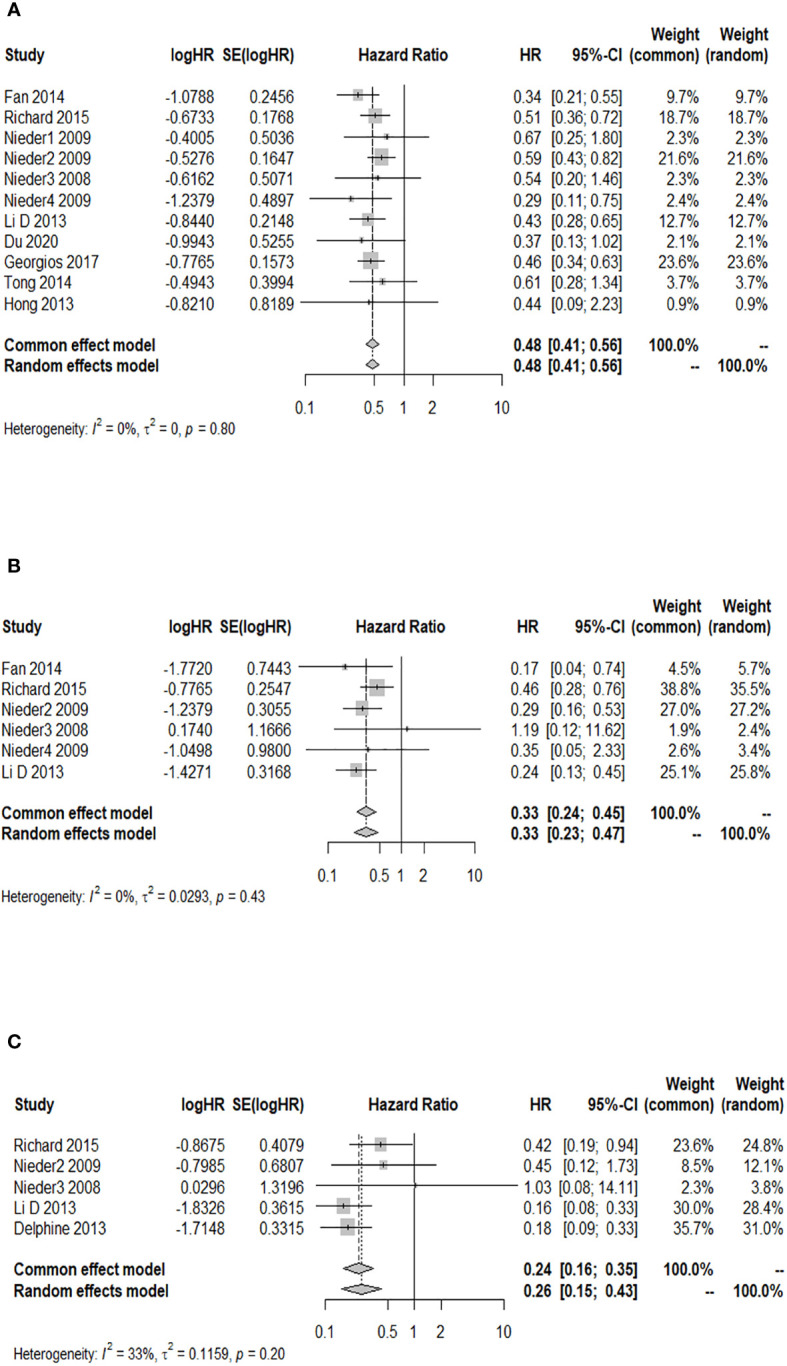
Forest plots of the association analysis between GPA and 6-month OS after WBRT in patients with BM. **(A)** Comparison of 6-month OS after WBRT in patients with BM of GPA 1.5–2.5 vs. 0–1. **(B)** Comparison of 6-month OS after WBRT in patients with BM of GPA 3 vs. 0–1. **(C)** Comparison of 6-month OS after WBRT in patients with BM of GPA 3.5–4 vs. 0–1.

A total of 11 cohort studies provided the 6-month OS rate after WBRT in patients with a GPA of 1.5–2.5 compared to a GPA of 0–1. The *I* ^2^ test revealed no significant heterogeneity among the 11 studies (*I* ^2 ^= 0.0%), and the fixed-effects model was used for the combined analysis. **Figure 3A** demonstrates a significantly higher 6-month OS rate in patients with a GPA of 1.5–2.5 than in the GPA of 0–1 group after WBRT (HR = 0.48; 95% CI: 0.41–0.56). The results of sensitivity analysis showed that (the combined HR ranged from 0.47 to 0.50), which indicated consistent results with the original results, so the original results were reliable (**Supplementary Figure 1**). Moreover, the funnel plot distribution appeared to be generally symmetrical (**Supplementary Figure 2**), and Egger’s test (*p* = 0.595) suggested the absence of potential publication bias. Six studies reported the 6-month OS rate after WBRT in patients with a GPA of 3 compared to a GPA of 0–1. The fixed-effects model was selected for meta-analysis due to homogeneity among these 6 studies (*I* ^2 ^= 0.0%). The 6-month OS rate was significantly higher in patients with a GPA of 3 than in the GPA of 0–1 group after WBRT (HR = 0.33; 95% CI: 0.24–0.45, **Figure 3B**). Additionally, a total of five studies reported the 6-month OS rate after WBRT in patients with a GPA of 3.5–4 and GPA of 0–1 (*I* ^2 ^= 33%). As shown in **Figure 3C**, the 6-month OS rate was significantly higher in patients with a GPA of 3.5–4 than in patients with a GPA of 0–1 after WBRT (HR = 0.24; 95% CI: 0.16–0.35).

### Association analysis between GPA and 12-month OS after WBRT in patients with BM

**Figure 4:**
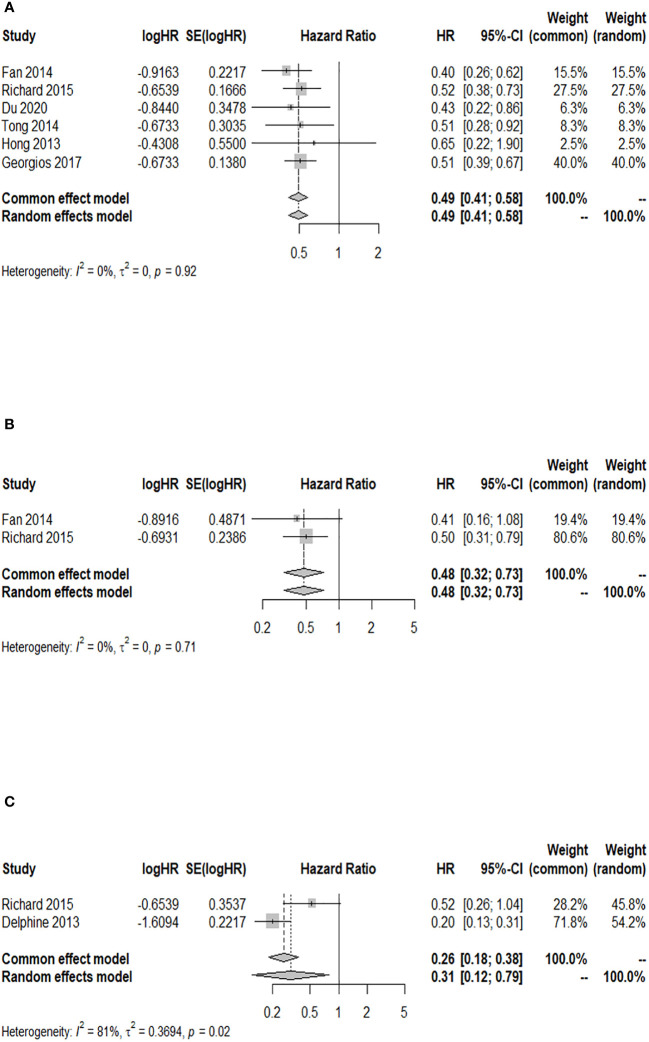
Forest plots of the association analysis between GPA and 12-month OS after WBRT in patients with BM. **(A)** Comparison of 12-month OS after WBRT in patients with BM of GPA 1.5–2.5 vs. 0–1. **(B)** Comparison of 12-month OS after WBRT in patients with BM of GPA 3 vs. 0–1. **(C)** Comparison of 12-month OS after WBRT in patients with BM of GPA 3.5–4 vs. 0–1.

A total of six studies provided the 12-month OS rate for patients with a GPA of 1.5–2.5 compared to a GPA of 0–1. There was no significant heterogeneity among these studies (*p* = 0.916, *I* ^2 ^= 0.0%), and the fixed-effects model was used for meta-analysis. **Figure 4A** illustrates that patients with a GPA of 1.5–2.5 had a significantly higher 12-month OS rate after WBRT than patients with GPA of 0–1 (HR = 0.49; 95% CI: 0.41–0.58). Two studies reported the 12-month OS rate after WBRT in patients with a GPA of 3.0 compared to a GPA of 0–1, and no significant heterogeneity was found (*p* = 0.714, *I* ^2 ^= 0.0%). The fixed-effects model was selected for the combined analysis. **Figure 4B** demonstrates that patients with a GPA of 3.0 had a significantly higher 12-month OS rate than those with a GPA of 0–1 (HR = 0.48; 95% CI: 0.32–0.73) after WBRT. Two articles provided the 12-month OS rate after WBRT in BM patients with a GPA of 3.5–4 vs. a GPA of 0-1, showing heterogeneity between the studies (*p* = 0.022, *I* ^2 ^= 80.9%). A random-effects model was used for the meta-analysis, as shown in **Figure 4C**. It revealed that patients with a GPA of 3.5–4 had a significantly higher 12-month OS rate after WBRT than patients with a GPA of 0–1 (HR = 0.31; 95% CI: 0.12–0.79).

### Association analysis between GPA and 24-month OS after WBRT in patients with BM

**Figure 5:**
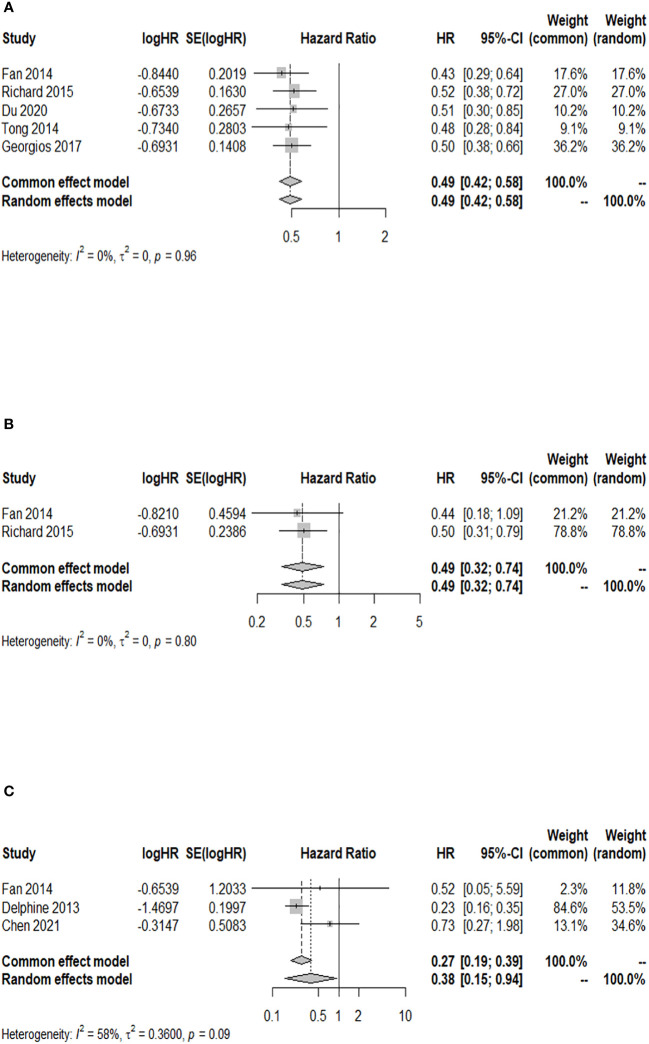
Forest plots of the association analysis between GPA and 24-month OS after WBRT in patients with BM. **(A)** Comparison of 24-month OS after WBRT in patients with BM of GPA 1.5–2.5 vs. 0–1. **(B)** Comparison of 24-month OS after WBRT in patients with BM of GPA 3 vs. 0–1. **(C)** Comparison of 24-month OS after WBRT in patients with BM of GPA 3.5–4 vs. 0–1.

Among the cohort studies included in the analysis, five studies reported the 24-month OS rate after WBRT in BM patients with a GPA of 1.5–2.5 compared to those with a GPA of 0–1, and there was no heterogeneity detected among the included studies (*I* ^2 ^= 0.0%). The results indicated a significant increase in the 24-month OS rate of BM patients with a GPA of 1.5–2.5 compared to those with a GPA of 0–1 after WBRT (HR = 0.49; 95% CI: 0.42–0.58, **Figure 5A**). Two of the included cohort studies reported the 24-month OS rate after WBRT in BM patients with a GPA of 3.0 compared to those with a GPA of 0–1. No heterogeneity was observed between these two studies (*I* ^2 ^= 0.0%), so a fixed-effects model was used. The 24-month OS rate for BM patients with a GPA of 3.0 was higher than that for BM patients with a GPA of 0–1 after WBRT, and the difference was statistically significant (HR = 0.49; 95% CI: 0.32–0.74, **Figure 5B**). Three of the included cohort studies reported the 24-month OS after WBRT in patients with a GPA of 3.5–4 compared to those with a GPA of 0–1, and heterogeneity was identified between these two studies (*p* = 0.092, *I* ^2 ^= 58.1%), so a random-effects model was chosen for meta-analysis. The results of the meta-analysis showed that at 24 months after WBRT, the OS rate for BM patients with a GPA of 3.5–4 was higher than that of patients with a GPA of 0–1, and the difference was statistically significant (HR = 0.38; 95% CI: 0.15–0.94, **Figure 5C**).

## Discussion

### The clinical implications of GPA in predicting the prognosis of BM patients after WBRT

The relevant study (33) has demonstrated that utilizing prognostic indices for BM can help identify favorable prognostic groups that may benefit from more intensified therapies while avoiding overtreatment in other groups. This approach facilitates the customization of treatment plans based on individual prognostic factors, thereby optimizing patient outcomes. In our study, we compared the OS rates at different time points after WBRT among patients with BM. We found that a higher GPA score was associated with a better prognosis.

We believe that our findings have the following clinical implications: The application prospects of the GPA model in personalized and precise treatment of BM are promising. By considering various factors such as age, Karnofsky Performance Status (KPS), number of BMs, and the presence of extracranial metastases, the GPA model can assist clinicians in evaluating the prognosis and survival outcomes of individual patients. Furthermore, it helps predict disease progression and survival time for BM patients.

This valuable information allows clinicians to stratify BM patients into different risk groups, enabling tailored treatment options and management strategies based on each patient’s specific needs. As a result, treatment outcomes and quality of life can potentially be improved. Additionally, regular monitoring of the GPA index allows clinicians to assess the effectiveness of treatment and track changes in the patient’s condition. This monitoring facilitates timely adjustments to the treatment plan, leading to better treatment outcomes.

### Practicability and limitations of WBRT and GPA model

Upon reviewing the relevant literature, it is apparent that there is limited research on the application of WBRT in the treatment of BM. In contrast, there has been a significant increase in studies investigating alternative treatments of BM such as SRS, targeted pharmaceutical interventions, and immunotherapy (62–67). Moreover, it remains unclear whether WBRT can improve the survival rate of patients (68). On the other hand, SRS not only enhances patients’ quality of life but also reduces the rate of cognitive decline (30). Additionally, targeted therapies have shown good efficacy with a relatively low incidence of side effects (69). The GPA model has demonstrated its effectiveness in predicting the prognosis of BM patients treated with WBRT or surgery, as confirmed by numerous clinical trials (38, 70–72). However, some clinical studies have indicated that the GPA model struggles to predict the changes in physical function and postoperative complications in BM patients treated with surgery. Moreover, the health status of the patient’s body and BM often fluctuate during the treatment process. Consequently, using a GPA model alone may not effectively predict the short-term prognosis of these patients. Evaluating the risks and benefits of surgical treatment for BM using the GPA model alone has yielded unsatisfactory results (71). This meta-analysis found that patients with BM who had a high GPA demonstrated significantly higher OS and a better prognostic status at the same time point. However, the available survival data at certain time points in this study were limited, and further investigation is needed to determine the value of GPA in evaluating the long-term prognostic survival of BM patients treated with WBRT.

### Comparison of commonly used prognostic assessment models

In clinical practice, it is crucial to select appropriate prognostic assessment models to prevent the abandonment of treatment due to inaccurate predictions (73). When comparing the more commonly applied prognostic assessment models of GPA, Recursive Partition Analysis (RPA), the Score Index for Radiosurgery in Brain Metastases (SIR), and the Basic Score for Brain Metastases (BSBM), it can be seen that all of these systems incorporate two factors: the Karnofsky score and extracranial metastatic status. Additionally, the control status of the primary tumor is considered in RPA, SIR, and BSBM (34, 36, 37). However, there is a certain subjectivity in the clinical evaluation of whether the primary tumor is controlled. Neil’s study compared the clinical utility of RPA and GPA in predicting the moderate prognosis of BM patients (74). The study revealed that RPA encountered difficulties in further stratifying patients’ prognoses, while GPA overcame this limitation by providing clinicians with a more refined scope for subjective assessment when selecting treatment plans. This could potentially explain why GPA was deemed more beneficial in clinical practice (74). It is worth noting that RPA and BSBM do not include the item of BM status, including both the number and volume, which can significantly influence patients’ prognostic survival time. The RPA, being the earliest prognostic scoring system utilized, has limited applicability in clinical practice due to the imbalanced distribution of patients across its classification levels (75). Moreover, the BSBM does not take into account the status of brain disease, specifically the absence of tumor number and tumor volume, as factors in its assessment (76). The SIR model is rarely used in clinical practice because it incorporates the volume of the largest lesion, which is typically estimated at the time of SRS (77). However, in reality, patients often do not have precise knowledge of the true volume of their lesions when selecting treatment methods. This limitation reduces the practicality of the SIR model. In comparison, the GPA model offers advantages in terms of objectivity, quantification, and practicality of prediction. Looking ahead, it is hoped that there will be simpler and more accurate models for predicting the prognosis of patients with BM to provide more beneficial treatment plans for precise clinical diagnosis and treatment.

### Heterogeneity analysis

The heterogeneity observed in this study may be attributed to the individualized treatment principles applied to patients with BM, resulting in variations in treatment approaches. Clinical trials have shown that patients treated with WBRT combined with other therapies had better prognostic survival than those who used WBRT alone (78). Furthermore, the prognosis of patients with BM often varies depending on the location of the primary tumor. Moreover, some studies provided limited survival data for patients, making it impossible to derive meaningful HRs. As a result, only a small number of eligible studies were included in the meta-analysis, leading to potential heterogeneity.

### Strengths and limitations of this study

There are some strengths in this meta-analysis: Firstly, the included patients were clearly diagnosed with BM, and their treatment methods were limited to WBRT. Our prognosis survival prediction of patients with BM by GPA was more accurate and reliable because the limitations of treatment minimized their influence on prognosis as a confounding factor. Secondly, the study encompassed a comprehensive range of original literature from multiple databases, which effectively mitigated selection bias, enhancing the robustness of the findings. In our study, we included a total of 1,797 patients with BM, and the evaluation results of the NOS scale indicated that the quality of the included studies was high, thus ensuring the reliability of our findings. Thirdly, there was minimal heterogeneity among the included studies, except for one specific individual outcome (the 12- and 24-month OS rate for patients with BM in GPA 3.5–4 and 0–1 groups). The observed heterogeneity in these studies was probably due to the relatively small number of eligible studies at these two time points. Moreover, the funnel plot and Egger’s test revealed little potential publication bias among the included studies. The results of the sensitivity analysis confirmed the robustness and reliability of the original conclusions, ensuring the validity of the analysis.

However, this study may have the following limitations: First, all 13 included studies were retrospective cohort studies, and the information bias might be due to the fact that patients with BM had deviations in the memory of their own conditions. Additionally, the samples of the included studies were sourced from various countries in Europe and Asia, which inevitably caused regional limitations. The differences in regional, cultural, and environmental factors among different countries can lead to variations in research results. Therefore, the regional limitations may introduce biases in the representativeness of our research samples and impact the external validity of the findings. Moreover, the number of patients meeting our requirements for individual outcome indicators was limited, and some individual research studies did not provide sufficient valid data.

## Conclusion

The prognosis of BM patients with high GPAs after WBRT was generally better than that of patients with low GPAs. The GPA model can be utilized to predict the prognosis of patients with BM after WBRT.

## Data availability statement

The datasets presented in this study can be found in online repositories. The names of the repository/repositories and accession number(s) can be found in the article/**Supplementary Material.**

## Author contributions

XG: Data curation, Formal analysis, Investigation, Methodology, Writing – original draft. CK: Conceptualization, Supervision, Validation, Writing – review & editing. JW: Conceptualization, Supervision, Validation, Writing – review & editing.
