# Supplementary figures and images for: The association between graded prognostic assessment and the prognosis of brain metastases after whole brain radiotherapy: a meta-analysis
Source: Front Oncol. 2024 Jan 9;13:1322262. doi: 10.3389/fonc.2023.1322262 (PMC10803601; doi:10.3389/fonc.2023.1322262)

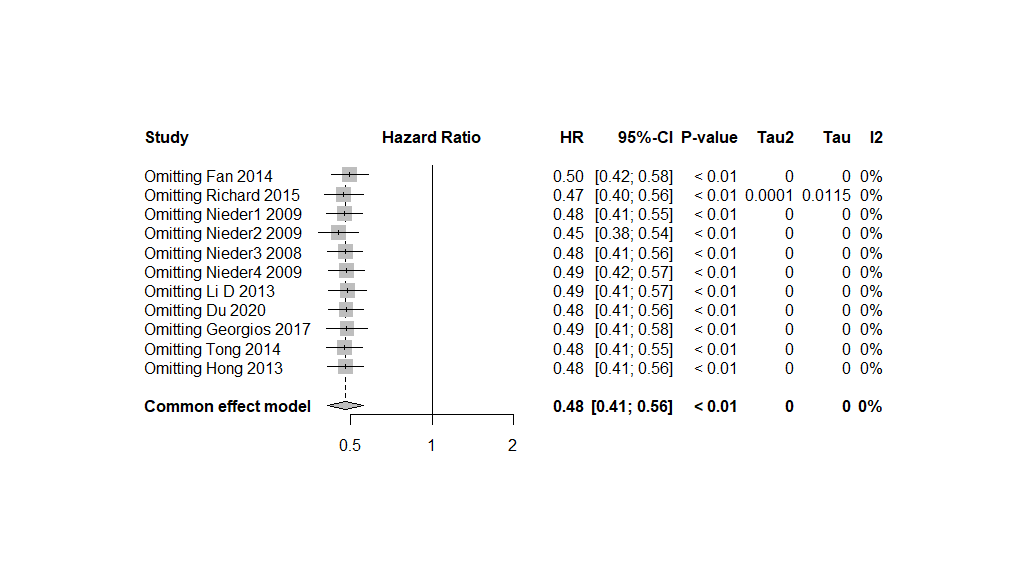

Supplement: Supplementary file 1 [file Image_1.tiff]

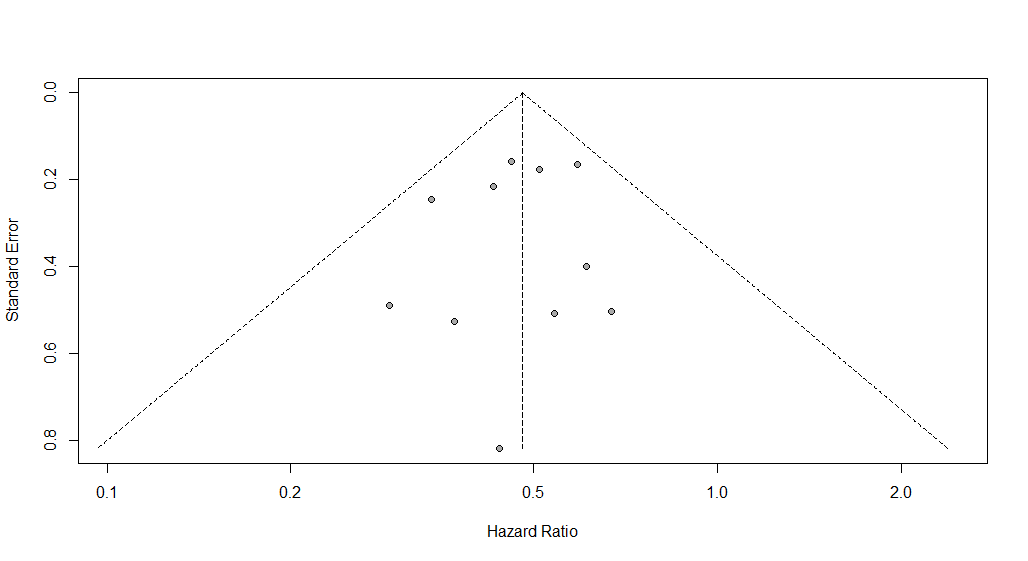

Supplement: Supplementary file 2 [file Image_2.tiff]
